# Dysregulation of the endothelin pathway in lymphangioleiomyomatosis with no direct effect on cell proliferation and migration

**DOI:** 10.1038/s41598-018-32795-3

**Published:** 2018-10-02

**Authors:** Nader Chebib, Fabienne Archer, Alexandra Bobet-Erny, Caroline Leroux, Vincent Cottin

**Affiliations:** 1 Université de Lyon, Université Claude Bernard Lyon 1, INRA, EPHE, IVPC, Viral Infections and Comparative Pathology, UMR754, F69007 Lyon, France; 2 Hospices Civils de Lyon, Groupement Hospitalier Est, Department of Respiratory Diseases, National Reference Center for Rare Pulmonary Diseases, Lyon, France

## Abstract

LAM is a rare low-grade metastasizing lung neoplasm. Inhibitors of mTOR improve clinical outcome of LAM patients by preventing loss of lung function. Nevertheless, other cell targets may be of interest for drug development. Therefore, we explored the potential role of EDN1 (endothelin) in LAM. We report an increased endothelin blood level in LAM patients as well as EDN1 overexpression and EDN1 receptor downregulation in LAM-derived primary cells and in TSC2^NEG^ cells mutated in TSC2. We evidenced EDN pathway dysregulation based on EDN1, EDNRA, EDNRB and ARRB1 mRNA expression in LAM-derived primary cells. We showed overexpression of EDN1 and ARRB1 mRNAs in TSC2^NEG^ cells; these cells lost their ability to respond to stimulation by endothelin. We analyzed the effects of endothelin receptor antagonists alone or in combination with rapamycin, an mTOR inhibitor, on proliferation and migration of LAM cells. Rapamycin treatment of TSC2^NEG^ cells significantly reduced cell proliferation or migration, while none of the tested inhibitors of EDN receptors impaired these functions. We showed that TSC2^NEG^ cells have acquired a transformed phenotype as showed by their ability to grow as spheroids in semi-solid medium and that unlike endothelin receptors antagonists, rapamycin reduced anchorage-independent cell growth and prevented expansion of TSC2^NEG^ spheroids.

## Introduction

Lymphangioleiomyomatosis (LAM) is a rare pulmonary disease mainly affecting young women^1^. LAM can occur as an isolated disorder, defined as sporadic LAM or in patients with tuberous sclerosis complex, a genetic disease characterized by mutations of the *TSC1* and *TSC2* (*Tuberous Sclerosis Complex*) tumor suppressor genes respectively encoding hamartin and tuberin^2^. LAM cells carry mutations of the *TSC2* gene^3^, inducing constitutive activation of the PI3K/Akt/mTOR pathway and LAM cell proliferation. LAM causes cystic destruction of the lungs and development of benign renal tumors or angiomyolipomas^1^. Two cell populations are present in LAM lesions: the myofibroblastic-like cells that express markers of smooth muscle cells and fibroblasts, such as α-smooth muscle actin (α-SMA), vimentin and desmin^4,5^ and the epithelioid-like cells that express melanocytic markers such as MLANA (Melan A) and proteins evidenced with HMB45 and PNL2 antibodies^5,6^. In LAM patients, circulating VEGF-D (Vascular Endothelial Growth Factor D) is increased in the blood and is associated with lymphangiogenesis, a major pathogenic mechanism in LAM progression^7,8^.

LAM is considered as a low-grade, destructive, metastasizing neoplasm^9^. Circulating LAM cells have been found in the blood, urine and chylous effusions^10,11^ of LAM patients. LAM cells invade organs through degradation of the extracellular matrix by metalloproteinases, similarly to metastatic cancer cells^12,13^.

Although mTOR inhibitors (everolimus, sirolimus) have been shown to improve clinical outcomes in preventing loss of lung function^14,15^ and have been approved to treat LAM, other pathways must be explored to improve patient treatment. In human cancer cells, high expression levels of EDN1 (Endothelin 1) and of endothelin receptors A and B (EDNRA and EDNRB) are associated with the increase of circulating VEGF and of microvessel density^16–19^. The EDN1/EDNR/ARRB1 (β Arrestin 1) pathway is implicated in cell proliferation, migration, invasion, survival and angiogenesis in several diseases, among them lung, ovary, prostate and breast cancers^20,21^. The development of endothelin receptor antagonists (ERAs) such as bosentan, a dual EDNRA and EDNRB receptor antagonist, or BQ123 targeting EDNRA, provided targeted treatments for pulmonary arterial hypertension and cancer^22–26^.

In this study, we explored the role of EDN1 and of its receptors in LAM-derived primary cells and in angiomyolipoma-derived cells lines. We report an increased blood level of endothelin in LAM patients as compared to controls, and the overexpression of EDN1 and downregulation of its receptors in LAM-derived primary cells as well as in TSC2^NEG^ cell lines. We analyzed the effects of ERAs, alone or in combination with rapamycin, on LAM cell proliferation and migration.

## Materials and Methods

### Cell lines

The 621-101 TSC2^NEG^ and 621-103 TSC2^POS^ cell lines (respectively named “TSC2^NEG^” and “TSC2^POS^” along our study) were generously provided by Pr E.P. Henske (Boston, United States)^27^. The TSC2^NEG^ cell line was derived from a renal angiomyolipoma of a LAM patient. They carry a missense mutation in exon 16 of the *TSC2* gene (G1832A) leading to a loss of heterozygosity. The TSC2^POS^ cell line has been developed by re-expression of normal *TSC2* gene in the 621-101 TSC2^NEG^ cells. These cell lines were cultured in DMEM medium (Sigma) supplemented with 10% inactivated fetal calf serum (Gibco), 100 U/mL penicillin (Sigma), 100 μg/mL streptomycin and with 50 μg/ml zeocin (Thermo Fisher) for the TSC2^POS^ cells to maintain the selective pressure for TSC2 expression.

Human primary PASMC (Pulmonary Artery Smooth Muscle Cells) (Lonza) were used as controls and maintained for a short time in culture as recommended.

### Lung-derived primary LAM cells

LAM pulmonary tissues and associated data from five patients (1300, 1444, 1720, 2634, 2749) were obtained from the Cardiobiotec biobank (CRB-HCL Hospices Civils de Lyon BB-0033-00046), a center for biological resources authorized by the French Ministry of Social Affairs and Health. All samples were collected and used in accordance with the ethical rules of the Biobank and in agreement with the French legislation. All patients signed a written informed consent. After surgical removal, tissue samples were immediately incubated in DMEM medium containing 100 U/mL penicillin, 100 μg/mL streptomycin, and 2.5 μg/mL amphotericin B (PAA). Tissues were dissociated in DMEM medium with 4.5 g/L glucose, 100 U/mL penicillin, 100 μg/mL streptomycin, 2.5 μg/mL amphotericin B, 2 mg/mL type Ia collagenase, 0.1 mg/mL soybean and 3 mg/mL elastase for 60 minutes at 37 °C under gentle agitation. After filtration on gauze, the suspension was centrifuged at 340 g for 10 minutes at 4 °C then cell pellets were cultured in DMEM/HAM’S F12 (50:50, V/V) medium (Gibco) supplemented with 10% inactivated fetal calf serum, 100 U/mL penicillin, 100 μg/mL streptomycin and 2.5 μg/mL amphotericin B.

### Phenotypic characterization of the LAM-derived cells

Cells fixed with ice-cold acetone were labeled overnight at 4 °C with mouse PNL2 (1/25, Diagnostic Biosystem, Mob421-05), mouse HMB45 (1/50, Diagnostic Biosystem, Mob079-05), mouse anti-desmin (1/100, Diagnostic Biosystem, Mob060-05), mouse anti-αSMA (1/1000, Sigma, A2527) and mouse anti-vimentin (1/200, Sigma, V2258) antibodies then incubated for 1 hour with donkey anti-Goat IgG-DyLight® 488, (1/500, Abcam, ab96931) or mouse anti-IgG-Alexa488 (1/500, Invitrogen A1101) antibodies. Cells were stained with 1 μg/mL DAPI (4′-6′ diamidino-2-phenylindole), observed with an “AxioImager Z1 epifluorescence” microscope (Zeiss) and analyzed with the “Zen software” (Zeiss).

### Circulating EDN1 and VEGF-D in LAM patients

Sera from 28 LAM and 12 control patients with unrelated disorders (6 multiple sclerosis, 6 myocardial infarction) were obtained from the Cardiobiotec biobank. EDN1 and VEGF-D were measured in the serum of LAM patients and controls using “solid-phase ELISA Quantikine Endothelin-1” and “VEGF-D immunoassay” (R&D systems) as recommended.

### Expression of EDNRA and EDNRB proteins

Total cellular proteins were extracted from cell pellets with 20 mM Tris pH 8; 10% glycerol; 150 mM NaCl; 1% Triton X-100; 5 mM EDTA; 1 mM Na_3_VO_4_; 1 mM PMSF; 10 µg/mL aprotinin; 10 µg/mL leupeptin, then homogenized with four sonication cycles (20 seconds at 300 W). EDNRA and ENRB receptors were detected on nitrocellulose membrane (Biorad), pre-incubated in TBST-milk (Tris pH 7.6, NaCl, 0.05% Tween 20, 5% non-fat dry milk), by overnight incubation at 4 °C with rabbit anti- EDNRA (SantaCruz, sc-33535) or rabbit anti-EDNRB (SantaCruz, sc-33537) antibodies followed by incubation with anti-rabbit peroxidase conjugated IgG antibodies (Sigma, A0545). The β-actin was detected with mouse anti-β-actin-peroxidase antibody (Sigma, A3854, 1/50 000). Immunoreactive bands were revealed using “West Pico chemiluminescent substrate” (Thermoscientific).

### Cell proliferation assay

TSC2^NEG^ and TSC2^POS^ cells were plated onto 96 wells plate at 20,000 cells/well and treated with 10 µM bosentan (Selleckchem), 10 µM BQ123 (Sigma-Aldrich), and/or 10 nM rapamycin (Calbiochem). Cellular proliferation was measured 1 and 3 days post treatment with the “CellTiter-Glo kit” (Promega) as recommended. Each experiment was done in triplicate.

### Cell migration assay

Forty-eight hours serum-deprived TSC2^NEG^ and TSC2^POS^ cells were plated in the top chamber of 8 μm pore diameter Fluoroblock cell culture inserts (Corning) in serum-free medium, while the bottom chamber was filled with DMEM medium supplemented with 10% fetal calf serum. Cells were cultivated with 10 nM rapamycin, 10 µM BQ123 or 10 µM bosentan. Fifteen hours after plating, cells were stained with calcein-AM fluorescent dye (Corning) and the fluorescence measured with a “Victor II” spectrophotometer (Wallac) using excitation filter at 488 nm and emission filter at 510 nm.

### Expression of EDN1, EDNRA, EDNRB and ARRB1 mRNAs

Total cellular RNAs were extracted with the “PureLink RNA mini kit” (Ambion), treated with the “Turbo DNA-free kit” (Ambion), and reverse transcribed with the “iScript cDNA synthesis” kit (Biorad). The mRNA expression was quantified in triplicate with the “PCR KAPA SYBR FAST qPCR” kit (Clinisciences) using 20 ng cDNA. Primer efficiency was determined by amplification of 10-fold dilutions of cDNAs. *PPIA* (*Peptidylprolyl Isomerase A*), *YWHAZ* (*Tyrosine 3 monooxygenase)* and *RPL32 (Ribosomal Protein L32) (*Table 1) have been selected as reference genes based on their stability in LAM samples evaluated with the geNorm software. Normalized relative expression was calculated with the 2^−ΔΔCq^ method^28^.

**Table 1:** PCR primers used in the study. *Reported in^23^.

Gene	Approved name	5′-3′ sequences (Forward Reverse)	Amplicon size (pb)	GenBank accession #
*YWHAZ*	Tyrosine 3-monooxygenase/tryptophan 5-monooxygenase activation protein zeta	F AACTCCCCTGAGAAAGCCTG	145	NC_019466
		R CCGATGTCCACAATGTCAAGTT		
*PPIA*	Peptidylprolyl isomerase A	F GTGCCAGGGTGGTGACTTC	200	XM_004013990
		R CCACATGCTTGCCATCCAAC-5′		
*RPL32*	Ribosomal protein L32	F GCACATGCTGCCCAGTGGCT	189	NM_000994.3
		R CGCAGCCTGGCATTGGGGTT		
*ARRB1*	Arrestin beta 1	F CGTGTGACTGGTGGGATGAA′	183	NM_004041.4
		R GCACAGAATTCCGCTTGTGG		
*EDN1*	Endothelin 1	F GCAGGAAAAGAACTCAGGGCTGAA	220	NM_001955.4
		R TGCCTTTCAGCTTGGGATCATGAAA		
*EDNRA*	Endothelin receptor A	F ACGAGATGGACAAGAACCGATGTGA	206	NM_001957
		R TCCGTTCATGGGGACCGAGGT		
*EDNRB*	Endothelin receptor B	F ACCTCAGCAGGATTCTGAAGCTCA	209	NM_000115
		R TCAAATGACTGGCACCAGCAGCA		
*MLANA*	Melan-A	F TGTGCCTTAACAAGAAGATGCCCAC	145	*NM_005511*
		R CTGCAGAACAGTCACCACCACCTT		
*PMEL*	Premelanosome protein	F CGCTGATCGTGGGCATCTTGC	136	NM_001200054
		R CGGGGTAGACGCAGCCAGTG		
*TYRP1*	Tyrosinase related protein 1	F ATGGCCAAGTCGGGAGTTTAGTGT	123	NM_000550
		R TCCATACTGCGTCTGGCACGAA		
*ACTA2*	Actin, alpha 2, smooth muscle, aorta	F TCCGCTGCCCAGAGACCCTG	146	NM_001141945
		R GGTGCCCCCTGATAGGACATTGTT		
*TSC2 Exon 40/41	TSC complex subunit 2	F ATGGAGGGCCTTGTGGACAC	249	NC_000016.10
		R CGGAGCCGCTTGATGTG		
*TSC2 Exon 6		F GGAGATGTAGATTCGGCGTC	222	
		R CTGCGGAGCTGAACTTAGG		

### *TSC2* mutations

Mutations in exons 40–41 region (nt 13535–13756, GenBank # NC_000016.10) and exon 6 splicing site (nt 46232–46500, GenBank # NC_000016.10) of the *TSC2* gene were screened in TSC2^NEG^, TSC2^POS^ cell lines and in LAM-derived primary cells as previously reported^29^. Genomic DNA was extracted with Quick DNA Universal Kit (Zymo Research) and amplified with the “KAPA HIFI Hotstart kit” (Kapa Biosystem). PCR products were purified with the “Zymoclean Gel DNA recovery” kit (Zymo Research), sequenced (GATC, France) and analyzed with the Vector NTI software (Invitrogen).

### Statistical analysis

Normalized gene expression was done with REST 2009 software (Qiagen). Statistical analysis was done with SPSS Statistics (Version 21, International Business Machines, USA) and PRISM software (GraphPad). Quantitative variables were compared with Mann-Whitney tests. All tests were done with a significance threshold of α = 0.05.

## Results

### Elevated levels of circulating EDN1 and VEGF-D in LAM patients

**Figure 1:**
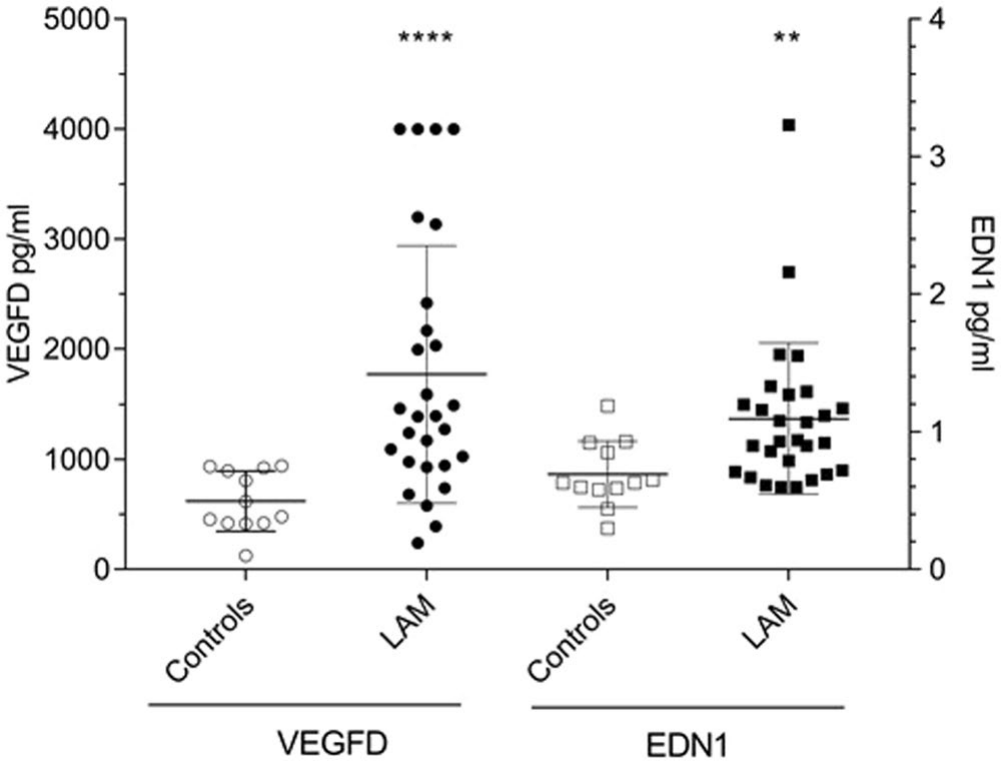
Circulating EDN1 and VEGF-D in LAM patients. Concentrations (pg/ml) of EDN1 and VEGF-D were measured by ELISA in control (n = 12) and LAM (n = 28) sera. P values calculated with the Mann-Whitney test; **p < 0.01; ****p < 0.0001.

EDN1 and VEGF-D levels were measured in sera obtained from 28 LAM patients and 12 female controls with unrelated disease. The circulating level of VEGF-D was significantly higher in LAM patients compared to controls (Fig. 1) with a VEGF-D level above 800 pg/ml in 82% (23/28) of LAM patients (and over 4000 pg/ml VEGF-D for four patients). The level of circulating EDN1 was significantly higher in LAM patients when compared to controls, suggesting a stimulation of the EDN1 production in LAM patients.

### Characterization of LAM- derived cells

**Figure 2:**
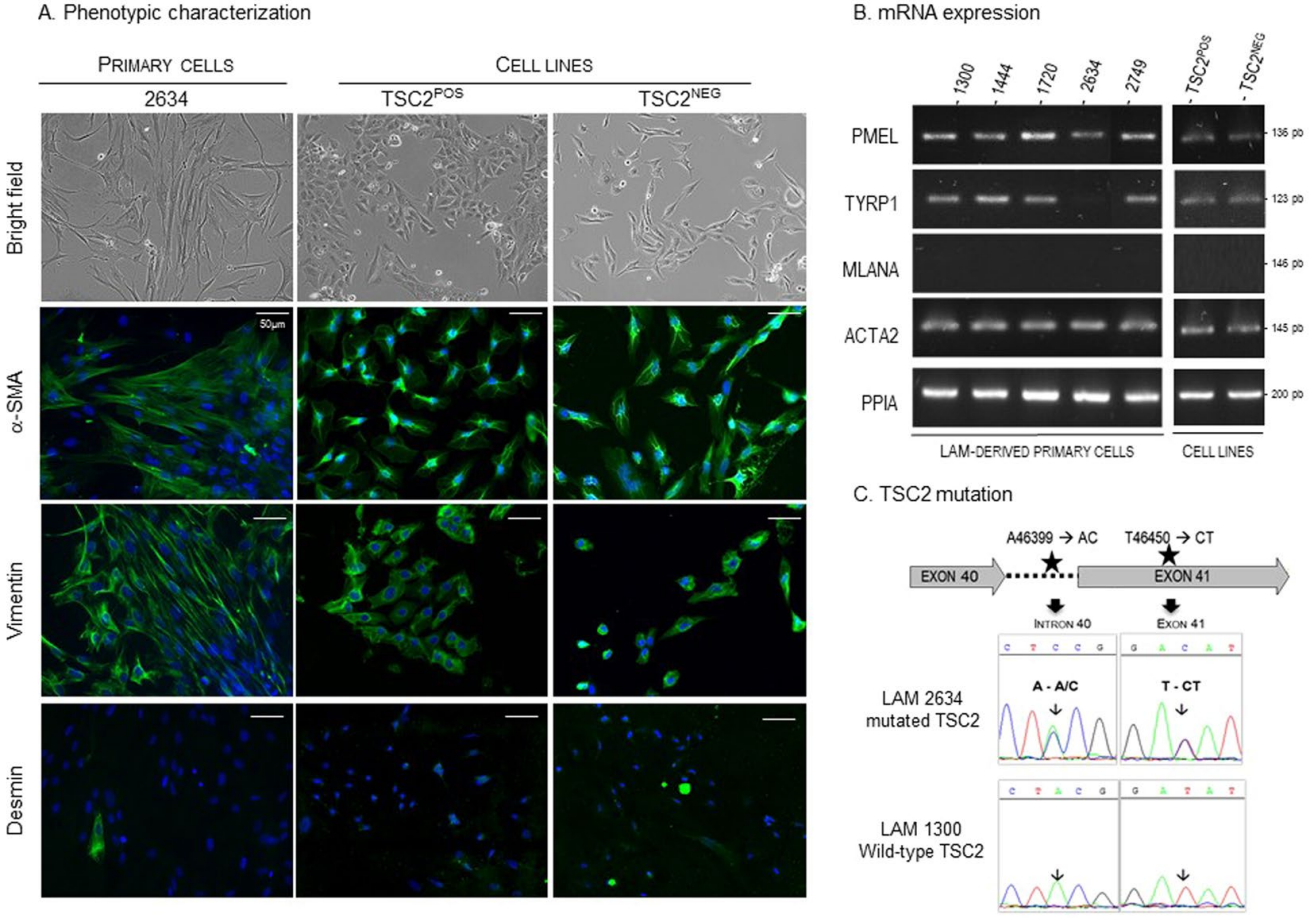
Characterization of lung-derived primary LAM cells, TSC2^NEG^ and TSC2^POS^ cells. (**A**) Morphology by phase contrast microscopy and expression of α-SMA, vimentin and desmin proteins (in green) in LAM cells derived from patient #2634, and in TSC2^NEG^ and TSC2^POS^ cells. Blue: nucleus stained with DAPI, magnification x400. (**B**) LAM primary cells and TSC2 cell lines expressed mRNAs of melanocytic (*PMEL*, *TYRP1*, *MLANA*) and smooth-muscle (*ACTA2*) genes. *PPIA* was used as reference gene. The figure was constructed from individual gels of *PMEL*, *TYRP1*, *MLANA*, *ACTA2 and PPIA* PCR products (see supplementals). (**C**) TSC2 point mutation in lung-derived primary LAM from patient #2634.

We generated lung-derived primary cells from five LAM patients. We observed mixed cell populations in the cultures, with mostly spindle-shaped cells and rare epithelioid-like cells (Fig. 2A), their respective proportion being patient-dependent. Primary LAM cells expressed α-SMA, vimentin and rarely desmin (Fig. 2A) and they were hardly positive for the melanocytic markers HMB45 and PNL2 (not shown). LAM cells expressed *PMEL* (pre-melanosome protein) but not *MLANA* (Melan-A) mRNAs (Fig. 2B). All but the cells derived from patient #2634 expressed *TYRP1* (Tyrosine related protein 1) mRNAs (Fig. 2B). Consistent with the protein expression, *ACTA2* (Actin alpha 2) mRNA was expressed in all cells. To summarize, the primary cells derived from LAM patients were consistent with myofibroblast-like cells expressing some of the melanocytic markers.

We morphologically characterized the TSC2^NEG^ and TSC2^POS^ cell lines; while the TSC2^NEG^ cells were spindle-shaped, the TSC2^POS^ cells were polygonal (Fig. 2A). TSC2^NEG^ and TSC2^POS^ cells expressed α-SMA, vimentin and barely desmin (Fig. 2A), but did not express melanocytic proteins as demonstrated by the absence of staining with HMB45 and PNL2 antibodies (not shown). However, both cell lines expressed mRNAs from melanocytic (*PMEL* and *TYRP1)* and myofibroblastic (*ACTA2)* genes (Fig. 2B). TSC2^NEG^ and TSC2^POS^ cell lines were therefore phenotypically compatible with LAM cells.

Because LAM is often associated with *TSC* mutations, pulmonary LAM cells were screened for mutations in DNA regions corresponding to exons 4041 and intron 5-exon 6 splicing region. We found two silent mutations (as compared to *TSC2* reference gene # NC_000016.10) in LAM patient #2634 with an A/C (nt 46399) transversion in the intronic region and a T/C (nt 46450) transversion in exon 41 (Fig. 2C).

### Expression of EDN1, EDNRA, EDNRB and β-arrestin (ARRB1) is dysregulated in LAM-derived cells

**Figure 3:**
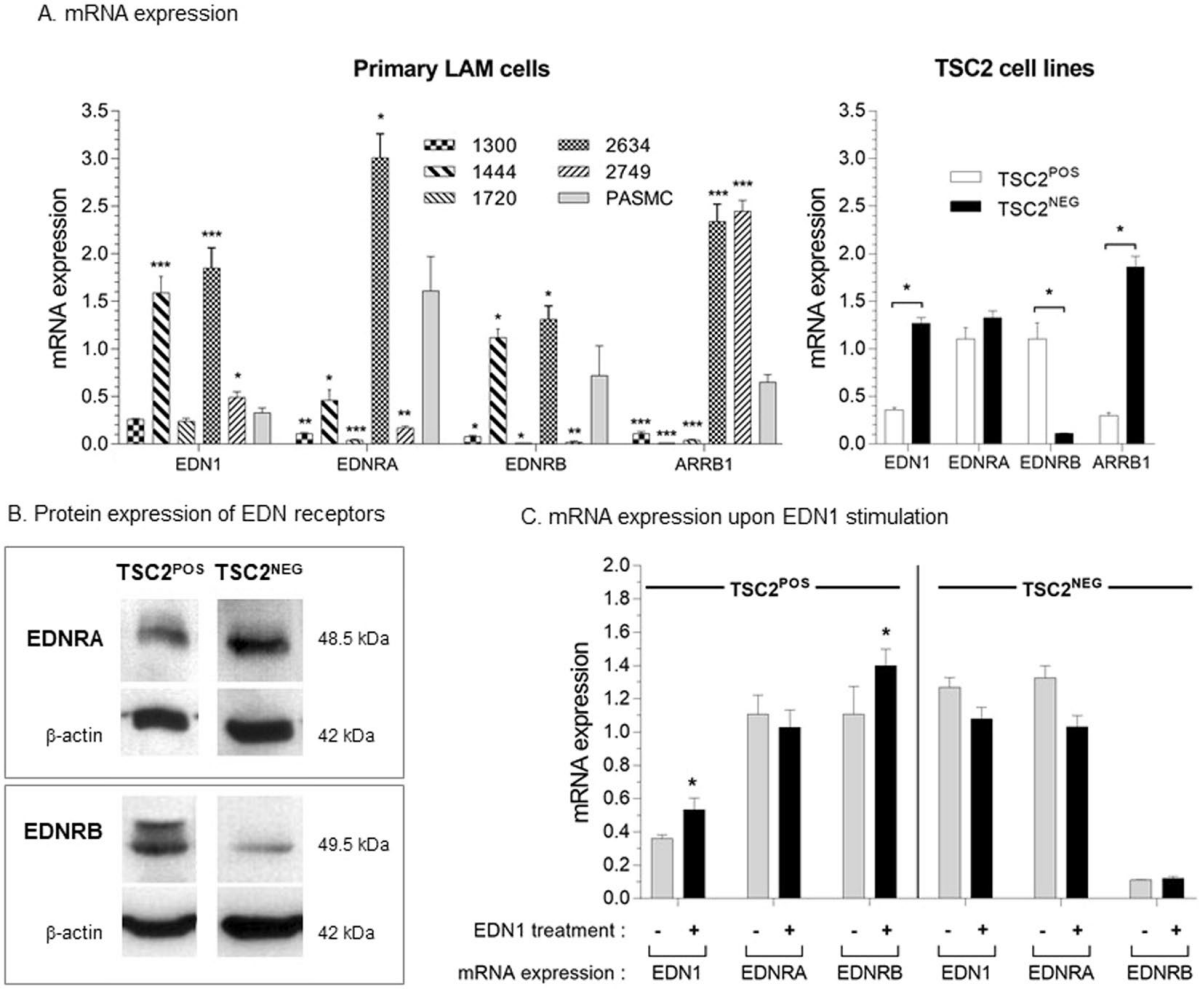
Dysregulation of expression of genes implicated in the endothelin pathway. (**A**) Expression of *EDN1*, *EDNRA*, *EDNRB* and *ARRB1* mRNAs has been semi-quantified by RT-qPCR in LAM-derived cells from patients 1300, 1444, 1720, 2634 and 2749 and PASMC (human primary Pulmonary Artery Smooth Muscle Cells) at early passages and in TSC2^NEG^ and TSC2^POS^ cell lines. The relative mRNA level is reported as mean +/− sem for each cell type. (**B**) Protein expression of EDNRA and EDNRB in TSC2^NEG^ and TSC2^POS^ cell lines. Figure was obtained from two individual membranes used to detect ENDRA or EDNRB. Each membrane was incubated with anti β-actin, to estimate the protein load (see supplementals). (**C**) EDN1, EDNRA and EDNRB gene expression was analyzed after treatment of TSC2^NEG^ and TSC2^POS^ cells with 100 nM endothelin for 24 h. *P* values were calculated with Mann-Whitney test.

We analyzed mRNA expression of EDN1, of its receptors (EDNRA and EDNRB), and of ARRB1 in lung-derived LAM cells and in TSC2^NEG^ (Fig. 3A). *EDN1*, *EDNRA*, *EDNRB* and *ARRB1* mRNAs were differentially expressed in the primary LAM cells derived from the five patients of our study. LAM cells derived from patient #2634 expressed high levels of *EDN1*, *EDNRA*, *EDNRB* and *ARRB1* mRNAs when compared to primary PASMC (Pulmonary Artery Smooth Muscle Cells) (Fig. 3A). Overexpression of *EDN1* mRNAs and of at least one of two EDNRs was found in three (#1444, #2634, #2749) out of the five lung-derived LAM cultures (Fig. 3A). Despite some heterogeneity of *EDN1*, *EDNRA*, *EDNRB* and *ARRB1* mRNA levels, we evidenced a transcriptional dysregulation of the EDN1 pathway in primary lung-derived LAM cells.

To investigate TSC2 involvement in EDN pathway dysregulation, we compared mRNA expression of *EDN1*, *EDNR* and *ARRB1* genes in TSC2^NEG^ and TSC^POS^ cell lines (Fig. 3A). The mRNA expression of *EDN1* and *ARRB1* was significantly higher (respectively 3.5 and 6.5-fold) while *EDNRB* mRNA expression was significantly lower (10 fold) in TSC^NEG^ as compared to TSC^POS^ cells (Fig. 3A); ENDRB protein expression was lower in TSC^NEG^ cells as compared to TSC^POS^ cells (Fig. 3A). Moreover, while EDNRA mRNAs were not differentially expressed in the two cell lines (Fig. 3A), higher levels of EDNRA proteins were detectable in TSC2^NEG^ cells as compared to TSC2^POS^ cells (Fig. 3B). Conversely, EDNRB proteins were expressed at a weaker level in TSC^NEG^ compared to TSC^POS^ cells (Fig. 3B).

Next, we tested the effect of endothelin stimulation by incubating both cell lines with 100 nM EDN1 for 24 hours. Following endothelin stimulation, expression of *EDN1* and *EDNRB* mRNAs significantly increased in TSC2^POS^ cells but stayed unchanged in TSC2^NEG^ cells (Fig. 3B) suggesting that TSC2^NEG^ cells lost their ability to respond to endothelin stimulation.

### Proliferation of TSC2^NEG^ cells upon EDNR and mTOR inhibitor treatment

**Figure 4:**
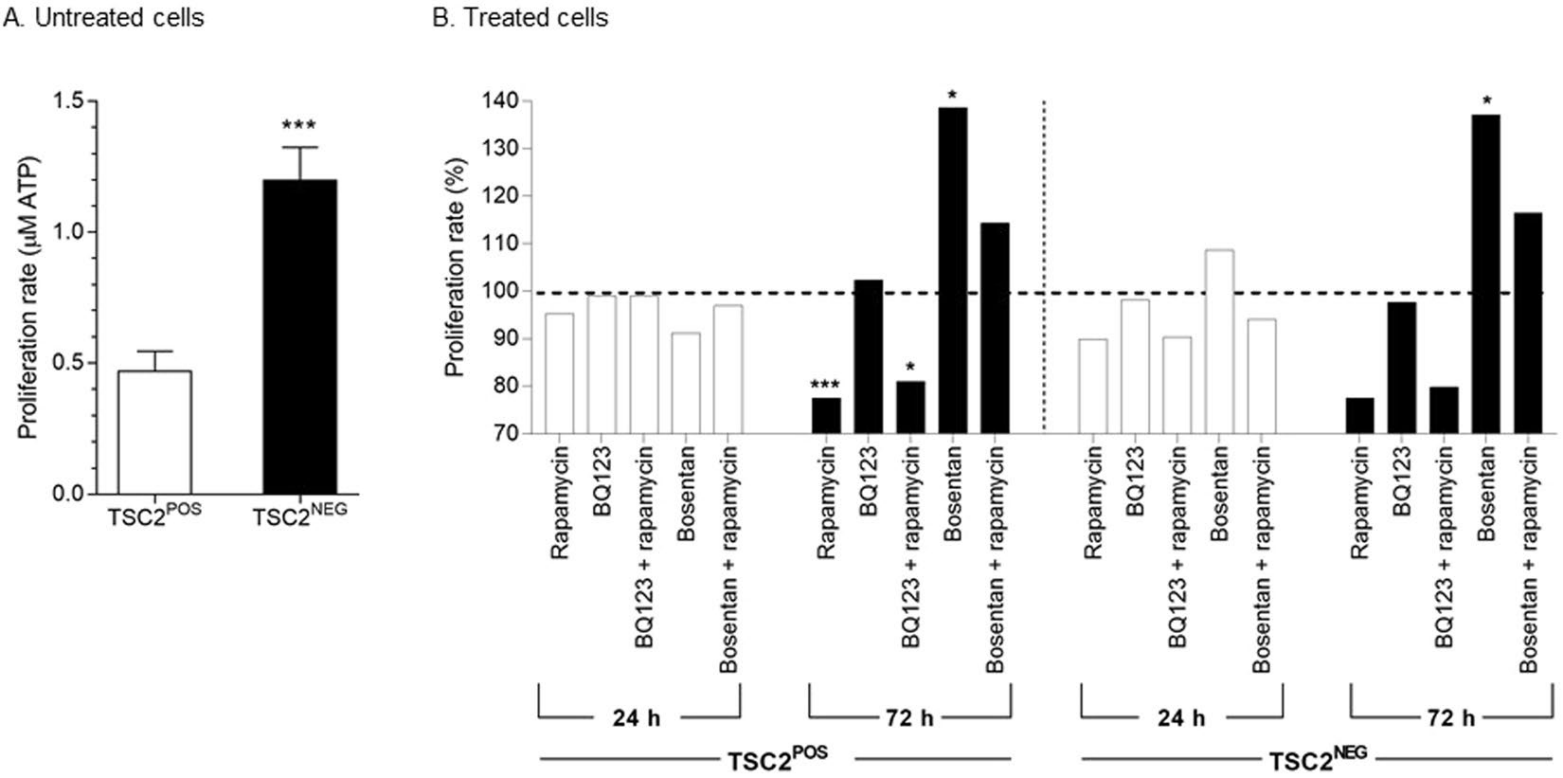
Proliferation of TSC2^NEG^ and TSC2^POS^ cells. (**A**) TSC2^NEG^ cells have a proliferative advantage when compared to TSC2^POS^ cells (***p < 0,001) (n = 3). Proliferation rate has been measured in triplicates and expressed in μM ATP. (**B**) TSC2^NEG^ and TSC2^POS^ cell lines were treated with 10 μM bosentan, or 10 μM BQ123 or 10 nM rapamycin, alone or in combination. Proliferation rate was measured 24 h and 72 h after inhibitor treatment and expressed as percentage of the control condition (DMSO or mock). Statistical analysis with Mann-Whitney test, *p < 0.1, ***p < 0.001.

We first showed that the TSC2^NEG^ cells had a significant proliferative advantage over the TSC2^POS^ cells (Fig. 4A). To investigate EDN pathway involvement in LAM, we tested the effect of ERA such as bosentan (EDNRA and EDNRB inhibitors) and BQ123 (EDNRA inhibitor), with or without rapamycin (mTOR inhibitor) on cell proliferation. Twenty-four hours of treatment with 10 µM bosentan, 10 µM BQ123 or 10 nM rapamycin alone or in combination did not significantly reduce TSC2^POS^ or TSC2^NEG^ cell proliferation (Fig. 4B). However, at 72 h post treatment, rapamycin was able to reduce the proliferation of both cell line, in a significant way for TSC2^NEG^ cells (Fig. 4B). Cell proliferation was also reduced when both cell lines were treated with a combination of BQ123 and rapamycin (Fig. 4B). Surprisingly, bosentan alone significantly increased TSC2^POS^ and TSC2^NEG^ proliferation (Fig. 4B). These results demonstrated the efficient inhibition of cell proliferation upon rapamycin treatment and suggested that inhibitor of EDNRA alone such as BQ123 do not alter proliferative rate of TSC2^NEG^ cells, while bosentan (targeting both EDNRA and EDNRB) activates cell proliferation.

### Migration of TSC2^NEG^ cells treated with inhibitors of EDNR or mTOR

**Figure 5:**
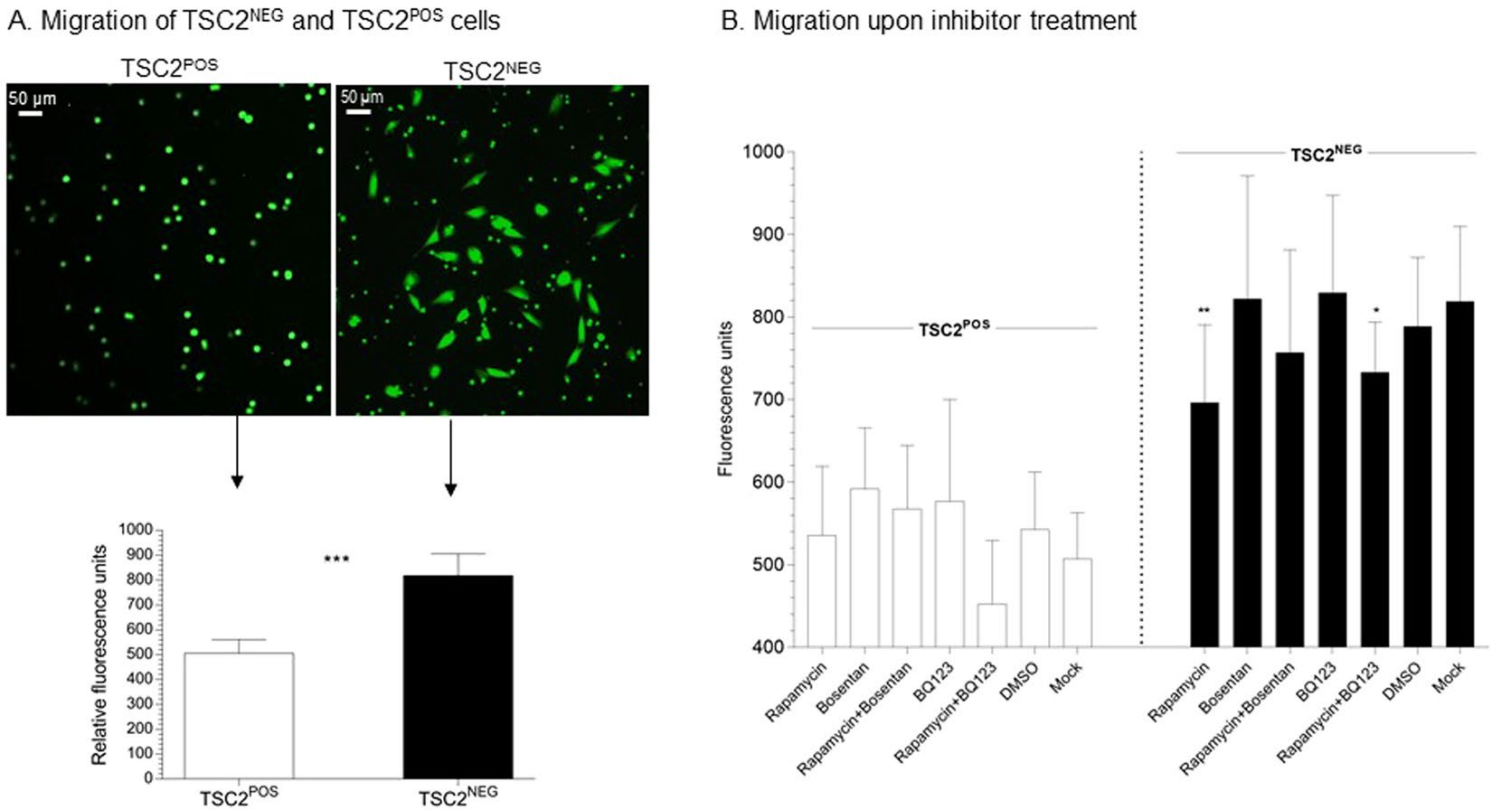
Migration properties of TSC2^NEG^. Cell migration has been determined by plating cells in Fluoroblock cell culture inserts, 8 μm pore diameter (Corning) and staining with fluorescent dye Calcein-AM 15 hours post treatment. (**A**) TSC2^NEG^ cells have higher migratory potential than TSC2^POS^ cells (****p < 0.0001, N = 12), as measured by the fluorescent cells on the lower side of the insert. (**B**) Cell migration was analyzed in presence of 10 nM rapamycin, 10 μM bosentan or 10 μM BQ123 alone or in combination (rapamycin + bosentan or rapamycin + BQ123). Rapamycin alone (**p < 0,01) or combined to BQ123 (*p < 0,05) significantly inhibited TSC2^NEG^ cell migration. Each condition was tested in four points, and experiments replicated 3 times. Statistical analyses were performed using the Mann Whitney test.

We first established that TSC2^NEG^ cells had a significant migration advantage over TSC2^POS^ cells (Fig. 5A). Using fluorescent labeling to follow migration, we showed that while TSC2^POS^ cells poorly migrated from the upper to the lower compartment of the insert, TSC2^NEG^ cells efficiently migrated as measured by the level of fluorescence signal (Fig. 5A). We then analyzed EDN involvement in migration of TSC2^POS^ and TSC2^NEG^ cells treated with endothelin receptor antagonists, alone or in combination with rapamycin. The addition of 10 μM bosentan or 10 μM BQ123 did not affect cellular migration of TSC2^NEG^ or TSC2^POS^ cells (Fig. 5B), suggesting that the EDN1 pathway was not implicated in migration. However, rapamycin alone or in combination with BQ123 significantly reduced migration of TSC2^NEG^ cells but not of TSC2^POS^ cells (Fig. 5B). These results suggest that unlike the EDN pathway, the mTOR pathway is implicated in LAM cell migration *in vitro*.

### TSC2^NEG^ cell growth is anchorage-independent and is reduced by rapamycin in combination to ERA

**Figure 6:**
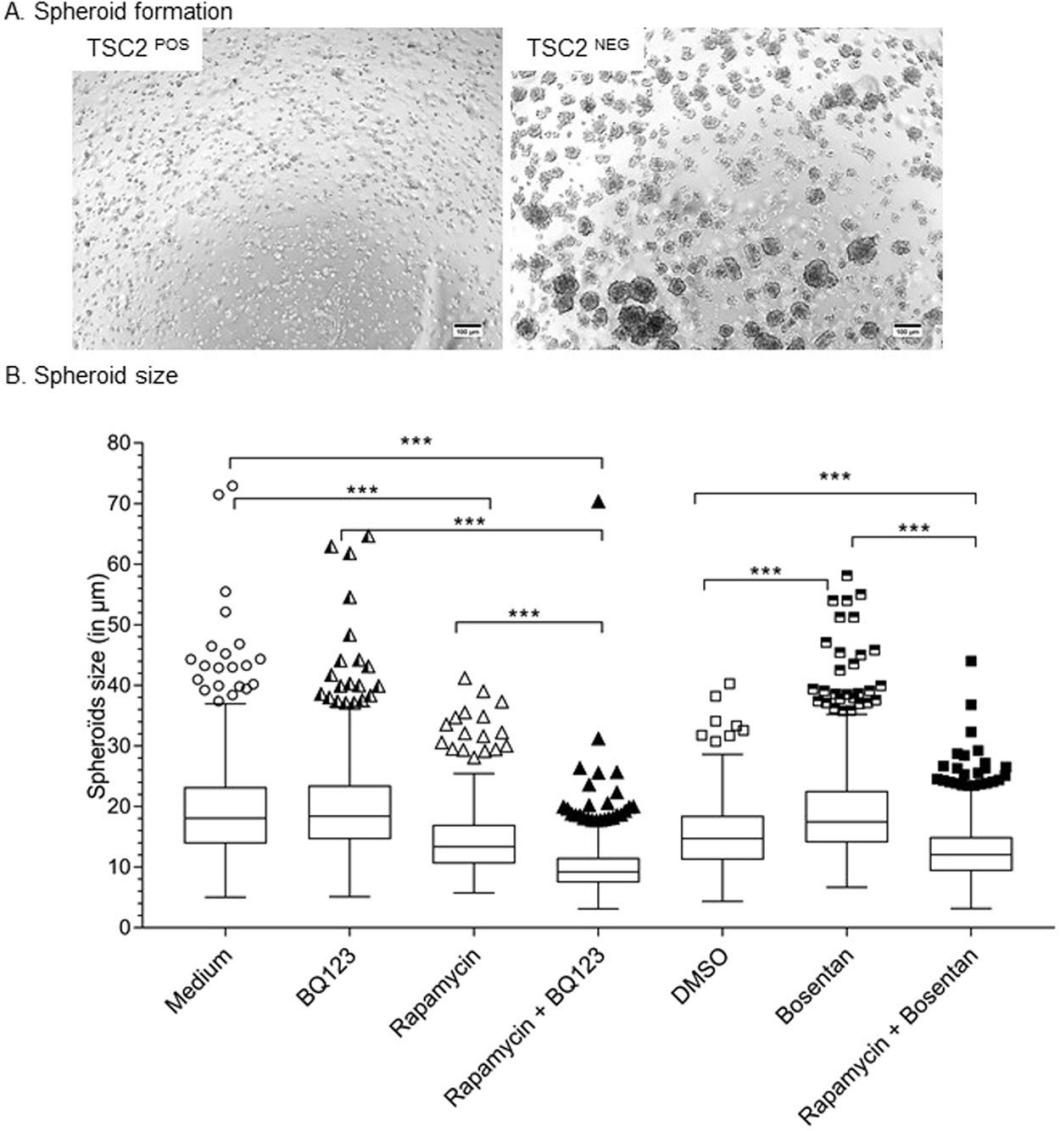
Anchorage- independent growth of TSC2^NEG^cells. TSC2^POS^ and TSC2^NEG^ cells were cultured in semi-solid medium for 6 days and monitored for the formation of spheroids. (**A**) Microscopic observation of cells growing in semi-solid medium. TSC2^NEG^ formed multiple spheroids while TSC2^POS^ were not able to grow without anchorage. (**B**) Cells were treated for 24 hours with 10 nM rapamycin, 10 μM BQ123, 10 μM bosentan alone or in dual combination and transferred in semi-solid medium. Spheroids formation was monitored at day 6. Spheroid size has been determined and compared between cells maintained in presence of inhibitors or in non-treated semi-solid medium (BQ123, rapamycin or rapamycin + BQ123) or semi-solid medium containing DMSO (bosentan or rapamycin + bosentan). Statistical analyses were performed using the Mann Whitney test (***p < 0.001).

When maintained in semi-solid medium, TSC2^NEG^ cells grew as spheroids, while TSC2^POS^ cells were unable to proliferate in 3D conditions (Fig. 6A), suggesting that TSC2^NEG^ cells had acquired a transformed or neoplastic phenotype. Interestingly, treatment with rapamycin alone or in combination with BQ123 or bosentan significantly reduced spheroids size when compared to untreated TSC^NEG^ cells (Fig. 6B). Endothelin receptor inhibitors alone (BQ123 or bosentan) were unable to decrease spheroid size (Fig. 6B) while bosentan alone had a reverse effect, favoring the growth of TSC2^NEG^ spheroids. In conclusion, while ERAs failed to alter anchorage-independent cell growth, rapamycin prevented expansion of TSC2^NEG^ spheroids.

## Discussion

LAM cells are characterized by increased proliferation associated to *TSC1* and *TSC2* mutations, leading to the activation of the mTOR pathway^30^. We hypothesized that dysregulation of the endothelin pathway could be implicated in LAM. The endothelin/β-arrestin/β-catenin pathway is activated in many cancers such as lung, ovary, prostate or breast cancers. It activates proliferation, migration, invasion and survival of cancer cells and stimulates angiogenesis^23^. To explore our hypothesis, we have successfully derived and characterized primary LAM cells that were compatible with the myofibroblast-like subpopulation of LAM, expressing α-smooth actin, desmin and vimentin. Lung-derived LAM primary cells hardly expressed melanocytic markers recognized by HMB45 and PNL2 antibodies but expressed *PMEL* and *TYRP1* mRNAs. The derivation of pulmonary LAM cells revealed to be challenging, as these cells proliferated slowly and were not easily maintained in culture. Despite their limited lifespan, they may be of interest as an *ex vivo* model of LAM. We studied the expression of key genes of the endothelin pathway as an insight of what occurs in LAM patients. We have analyzed the expression of EDN1 and of its receptors EDNRA and EDNRB in primary LAM cells as well as in a *TSC2* mutated cell line derived from an angiomyolipoma. Interestingly, mRNA expression of EDN1, EDNRA and EDNRB genes was dysregulated. The TSC2^NEG^ cell line and most primary LAM cells expressed high levels of *EDN1* and *ARRB1* mRNAs and a low level of *EDNRB* mRNAs when compared to control cells. Unlike TSC2^POS^ cells, endothelin stimulation of TSC2^NEG^ cells did not affect *EDN1*, *EDNRA* or *EDNRB* mRNA expression, suggesting the loss of positive feedback, possibly due to an independent activation of the EDN1 pathway in these cells.

To investigate the role of these dysregulations in LAM, we studied the effect of EDN receptor inhibitors, combined or not with rapamycin in the LAM cell lines. Proliferation, migration and anchorage-independent growth of TSC2^NEG^ were not modified in presence of BQ123, an inhibitor of EDNRA; surprisingly bosentan, which targets EDNRA and EDNRB, activated cell proliferation. However, we showed that rapamycin was an inhibitor of TSC2^NEG^ cell migration and anchorage-independent growth.

Despite the expression of EDN in LAM cells, we showed that the EDN1 pathway was not implicated in cellular proliferation or migration or anchorage-independent growth, as demonstrated by the lack of inhibitory effects of ERAs such as BQ123. These results are in contrast with the beneficial effects of ERAs with anti-proliferative action on arterial smooth muscle cells in other diseases such as pulmonary arterial hypertension, where EDN1 is expressed in the pulmonary vasculature^31–33^.

We have shown that TSC2^NEG^ were highly proliferating and migrating cells. It has been reported that TSC2 controls cell migration through its N-terminus and that RhoA activity is necessary^34^. TSC2^POS^ control cells used in this study have been generated by re-establishment of normal *TSC2* gene expression in the TSC2^NEG^ cell line *via* a viral vector. The TSC2^POS^ cells that have regained *TSC2* expression are characterized by lower proliferation rate, no migration and no anchorage-independent growth; the restoration of *TSC2* expression in patients carrying mutations could open to new therapeutic approaches.

Randomized controlled trials have shown that mTOR inhibitors such as sirolimus and everolimus stabilize lung function and reduce angiomyolipoma size in LAM patients^15,35^. Our study is the first report on the inhibitory effect of rapamycin on cellular migration of TSC2-deficient cells. Apart from its effect on cell proliferation, mTOR inhibition may control cell migration in LAM patients; this could participate to the beneficial effect of mTOR inhibitors in LAM patients.

To summarize our results, we have established that the EDN1 pathway is dysregulated in TSC2-deficient cell lines as well as lung-derived primary LAM cells associated with high levels of *EDN* and *ARRB1* mRNA. While targeting EDN receptors with existing antagonists did not prevent proliferation, migration or anchorage-independent growth of LAM cells, β-arrestin 1 could be considered as an alternative target in LAM patients. β-arrestin 1 is a scaffold protein interacting with G protein-coupled receptors and is a major effector of the EDN1 pathway. The binding of EDN1 to EDNRA allows the activation and recruitment of β-arrestin 1 and the formation of β-arrestin 1 and β-catenin complexes^36^ that translocate to the nucleus and activate *EDN1* transcription^21,37–39^. Barbadin, a β-arrestin inhibitor, has been recently reported^40^ and could help to decipher the role of ARRB1 in LAM as a potential target for new therapeutic tools.

Interestingly, we have shown that rapamycin has an inhibitory effect on LAM cell migration and anchorage-independent growth; this may be of importance in understanding the beneficial effect of mTOR inhibitors in LAM patients.

## Electronic supplementary material

SUPPLEMENTALS
