# Supplementary material for: Dysregulation of the endothelin pathway in lymphangioleiomyomatosis with no direct effect on cell proliferation and migration
Source: Sci Rep. 2018 Oct 2;8:14698. doi: 10.1038/s41598-018-32795-3 (PMC6168484; doi:10.1038/s41598-018-32795-3)

Dysregulation of the endothelin pathway in lymphangioleiomyomatosis with no direct effect on cell proliferation and migration

Nader Chebib, Fabienne Archer , Alexandra Bobet-Erny, Caroline Leroux, Vincent Cottin

**Figure S1. The figure presents the agarose gels used to construct fig 2B showing the migration of the amplicons.**

The "LAM-derived primary cells" panel has been reconstructed from the following gel captures:


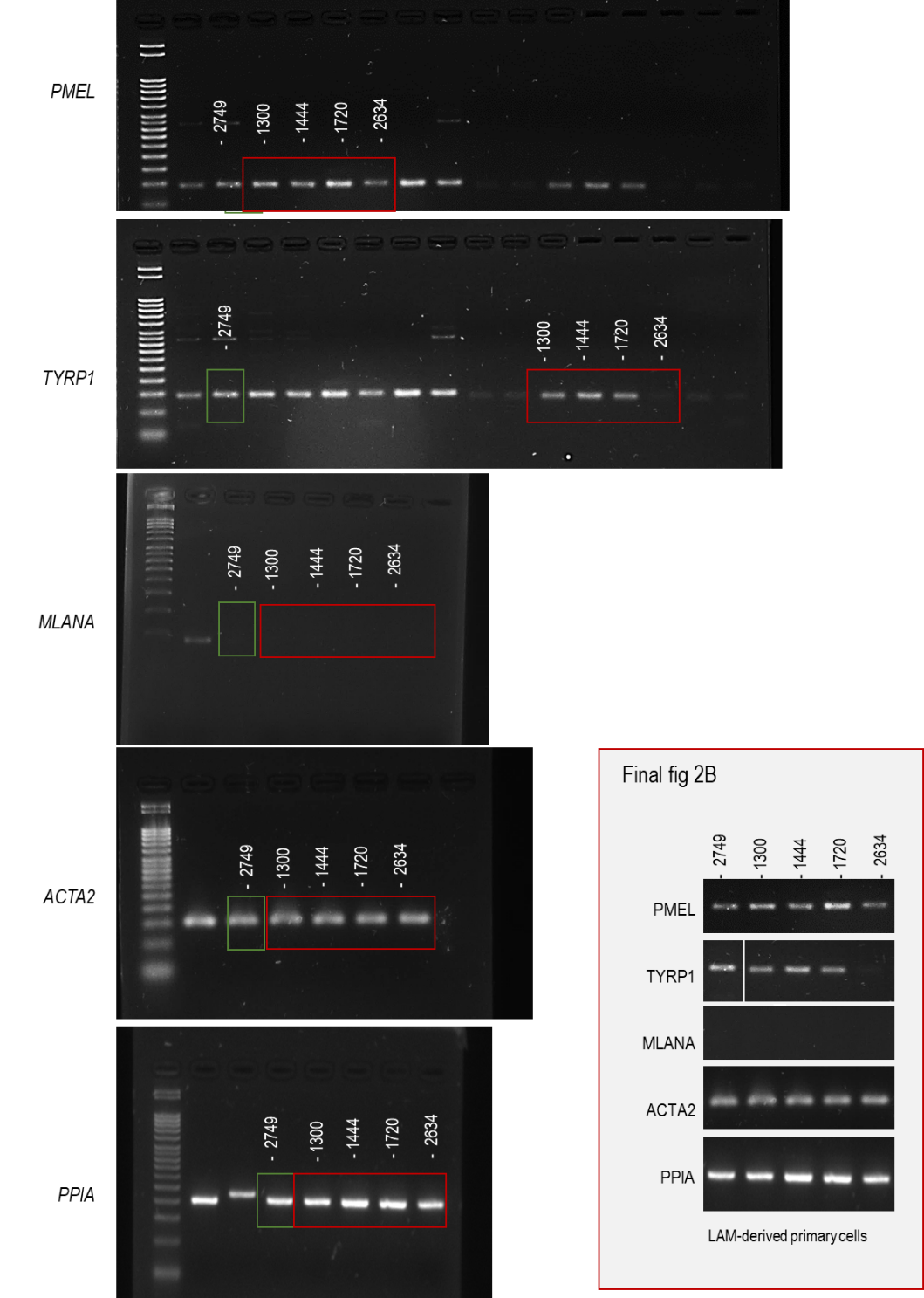


The "cell lines" panel has been reconstructed from the following gel captures:


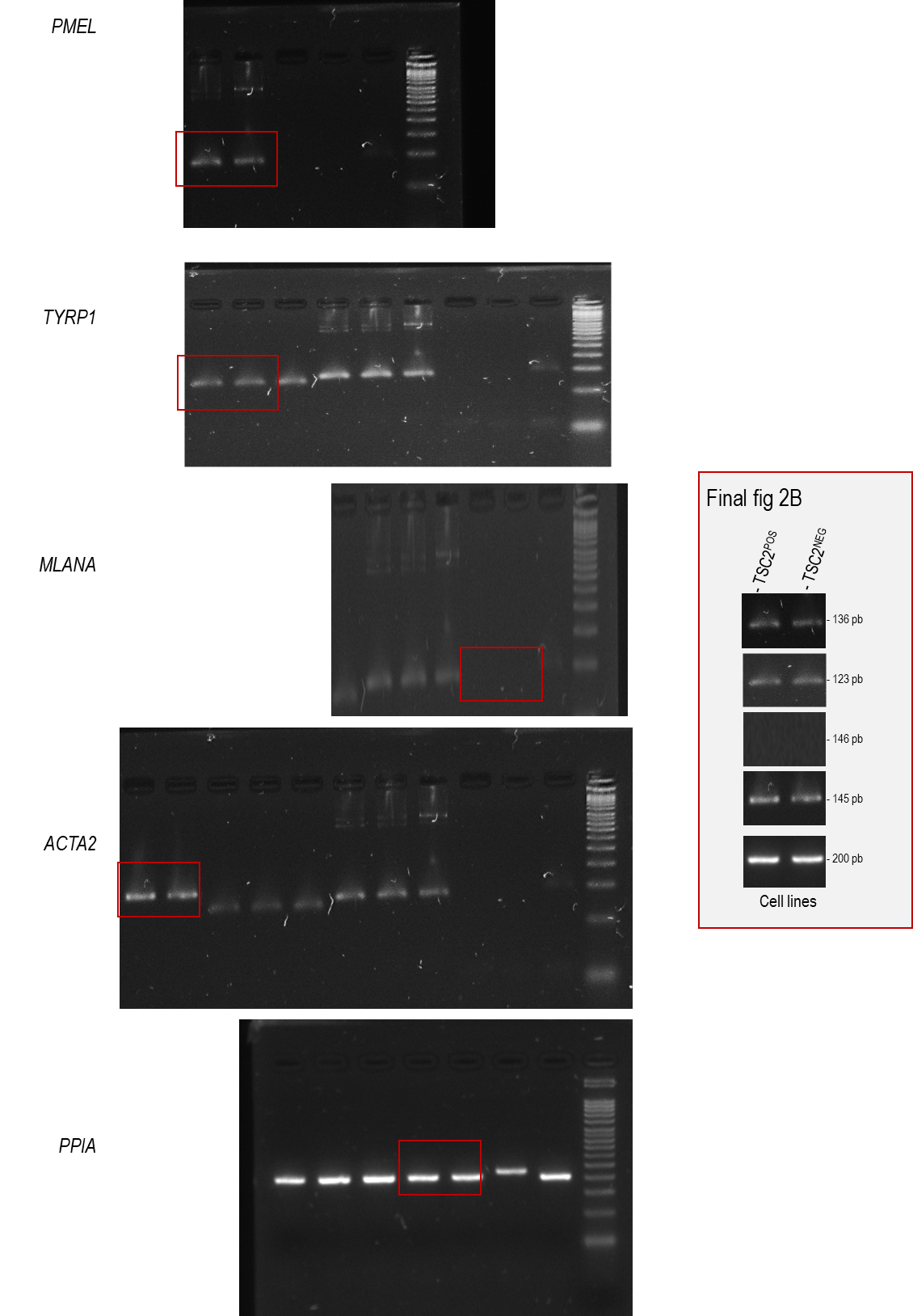


**Figure S2. The figure presents the immunoblots used to construct figure 3B**


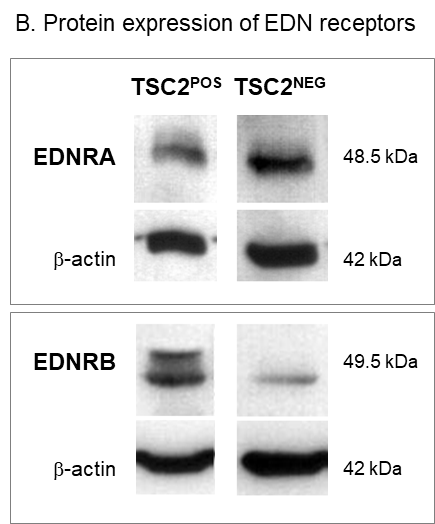


Figure 3B has been reconstructed from the following immunoblot. "621-101" and "621-103" lane are respectively named TSC2^NEG^ and TSC2^POS^ cell in the paper, as writtent in the Mat and Meth section. After detection of EDNRA (A) or EDNRB (B), the corresponding membrane have been blotted with antibodies directed against β-actin, to control the protein load


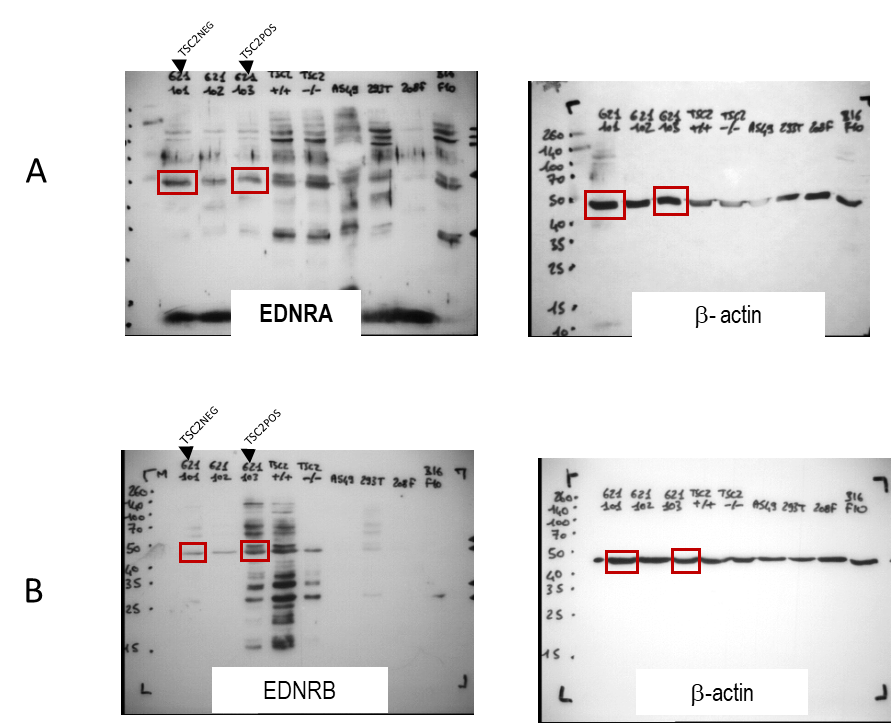

Supplement: Supplementary file 1 — SUPPLEMENTALS [file 41598_2018_32795_MOESM1_ESM.docx]
